# Comparison of the Early Clinical Outcomes between Combined Small-Incision Lenticule Extraction and Collagen Cross-Linking versus SMILE for Myopia

**DOI:** 10.1155/2016/2672980

**Published:** 2016-04-27

**Authors:** Alex L. K. Ng, Tommy C. Y. Chan, George P. M. Cheng, Vishal Jhanji, Cong Ye, Victor C. P. Woo, Jimmy S. M. Lai

**Affiliations:** ^1^Department of Ophthalmology, LKS Faculty of Medicine, The University of Hong Kong, Pokfulam, Hong Kong; ^2^Department of Ophthalmology, Hong Kong Eye Hospital, Kowloon, Hong Kong; ^3^Department of Ophthalmology and Visual Sciences, The Chinese University of Hong Kong, Kowloon, Hong Kong; ^4^Hong Kong Laser Eye Center, Kowloon, Hong Kong

## Abstract

*Background*. To compare the early outcome of combined SMILE and collagen crosslinking (SMILE Xtra) with SMILE.* Method*. Prospective, comparative interventional study of 21 eyes receiving SMILE Xtra using a low energy protocol and 32 control eyes receiving SMILE only. The outcomes were compared at 1, 3, and 6 months postoperatively.* Results*. Both groups had myopia with spherical equivalent refraction (SEQ) > 4.00 D. The SMILE Xtra group had thinner preoperative central corneal thickness and residual stromal bed thickness (*p* < 0.021). At 6 months, no eyes lost more than 1 line in corrected distance visual acuity. The safety index was 0.96 ± 0.06 and 1.00 ± 0.00 in SMILE Xtra and control, respectively (*p* < 0.001). 89% and 94% of eyes were within ±0.50 D of target refraction, respectively, with the mean error in SEQ correction being −0.17 ± 0.26 D for SMILE Xtra and +0.03 ± 0.25 D for control (*p* = 0.021). The efficacy index was 0.88 ± 0.13 and 0.97 ± 0.06, respectively (*p* = 0.005).* Conclusion*. SMILE Xtra had good overall safety profile and predictability at 6 months. However, when compared with control, the safety index and efficacy index were statistically significantly lower in the early postoperative period.

## 1. Introduction

Small-incision lenticule extraction (SMILE) is a recently developed corneal refractive procedure that has been shown to be safe and effective in correcting myopia [1–5]. The procedure involves cutting a small lenticule in the corneal stroma with a femtosecond laser, which is subsequently removed through a 2 to 3 mm small incision. Compared with laser in situ keratomileusis (LASIK), where a flap is created then lifted, SMILE is a flapless procedure and avoids the flap-related complications seen in LASIK surgery. It was shown in a randomized study that SMILE had comparable outcome with femtosecond-assisted LASIK [6–8].

In LASIK, a rare but devastating complication is iatrogenic corneal ectasia, where there is progressive corneal steepening and thinning resulting in increasing myopia and astigmatism and decrease in visual acuity. Although post-LASIK ectasia can now be effectively treated with crosslinking [9], advanced cases may still require keratoplasty. The main risk factors for developing post-LASIK ectasia include preexisting forme fruste keratoconus and a thin residual stromal bed (RSB) thickness [10, 11]. Factors that contribute to a thin RSB thickness include thin preoperation corneal thickness, correction of high myopia, and a thick flap. Therefore, careful preoperative patient selection is most important in the prevention of post-LASIK ectasia. As post-LASIK ectasia is thought to be related to the weakened biomechanical properties in post-LASIK eyes, prophylactic strengthening of the cornea with simultaneous cross-linking (LASIK Xtra) has been increasingly used in recent years [12–15], particularly in patients with young age, high myopia or high hyperopia, thin corneal thickness, and thin RSB thickness. The development of SMILE aimed for a biomechanically stronger cornea by preserving the integrity of the anterior cornea due to its flapless nature. Although large case series have demonstrated an excellent safety profile of SMILE [2], corneal ectasia after SMILE in eyes with preexisting forme fruste keratoconus has been reported [16, 17]. Combining collagen cross-linking with SMILE (SMILE Xtra) will have the potential advantage of restrengthening the cornea, which theoretically can reduce the risk of ectasia development and also regression of myopia. The purpose of this study is to compare the 6-month clinical outcome of a series of SMILE Xtra cases using a low energy protocol with SMILE alone.

## 2. Materials and Methods

A total of 21 consecutive eyes from 12 patients who have undergone SMILE Xtra between January and March 2015 at a private ophthalmic center in Hong Kong were recruited prospectively. A random sample of 32 eyes from 18 patients receiving SMILE alone (without CXL) during the same period were also recruited to serve as control. Inclusion criteria of the study included moderate to high myopia with baseline spherical equivalent refraction (SEQ) higher than 4 diopters, and all eyes had a plano target refraction. All patients had signed an informed consent before the refractive procedure. The study was approved by the Institutional Review Board of the University of Hong Kong/Hospital Authority Hong Kong West Cluster (Reference number: UW 15-226) and adhered to the tenets of the Declaration of Helsinki.

All patients underwent complete ophthalmic evaluation before the procedure and had no ocular comorbidities other than myopia and myopic astigmatism. All patients had preoperative CDVA of 20/20 or better. The ectasia risk factor score was also calculated based on the system proposed by Randleman, which took topographic pattern, age, CCT, RSB thickness, and SEQ into consideration [11]. The additional option of SMILE Xtra was offered to patients with a higher ectasia risk score. Although this scoring system was developed for LASIK, it was applied here given that a modified version does not yet exist for SMILE. Exclusion criteria for SMILE procedure included unstable refraction, topographic evidence of forme fruste keratoconus, significant ocular diseases, previous corneal surgery, autoimmune diseases or medications that affect wound healing, and pregnant or lactating patients.

### 2.1. Operative Procedures

The same surgeon (GC) performed all the surgery under topical anesthesia. SMILE was performed using the 500-kHz VisuMax femtosecond laser (Carl Zeiss Meditec, Jena, Germany) using an established technique [4]. The following parameters were used: cap thickness, 110–120 *μ*m; cap diameter, 7.0–7.5 mm; lenticule diameter, 6.0–6.5 mm with a transition zone of 0.1 mm; cut energy, 1.4 *μ*J; spot and tracking distance, 2.0–3.0 *μ*m. The back of the intrastromal lenticule was created from periphery to center of the cornea. The anterior lamellar cut was subsequently created from center to periphery of the cornea, which extended toward the surface to create a 2 mm incision located at 12 o'clock position (for left eye) or 10 o'clock position (for right eye), from which the stromal lenticule was extracted. A thin blunt spatula was used to separate the lenticule, which was then grasped with a pair of forceps and removed. The corneal interface was flushed with balanced salt solution. The same normogram was used for both groups. For cases with combined cross-linking, after lenticule removal, 0.22% riboflavin with saline (VibeX Xtra, Avedro) was instilled through the small incision into the interface and allowed for a soak time for 45 seconds. An additional 2 mm incision at the opposite site was created to allow for complete irrigation of the riboflavin solution. This was followed by ultraviolet A irradiation at 18 mW/cm^2^ for 45 seconds (total energy: 0.8 J/cm^2^) using the CXL-365 variosystem (Schwind, Germany).

Postoperative medications included topical tobramycin 0.3% and dexamethasone 0.1% ophthalmic suspension four times a day for 1 week. Preservative-free artificial teardrops were used for 3 months postoperatively. All patients were examined preoperatively and at postoperative day 1, week 1, month 1, month 3, and month 6. Assessments included monocular uncorrected distance visual acuity (UDVA) and CDVA, manifest refraction, and slit-lamp and fundus examination. Corneal thickness was measured with a scanning-slit topography (Orbscan; Bausch & Lomb, Rochester, NY). Efficacy index was calculated as the ratio of postoperative UDVA over preoperative CDVA and safety index was determined as the ratio of postoperative CDVA over preoperative CDVA. Outcome measures included postoperative UDVA, CDVA, corneal thickness, efficacy index, safety index, and the predictability of the correction. Any complications arising from the procedure were reported.

### 2.2. Statistical Analysis

Statistical analysis was performed using SPSS version 21 (IBM, Chicago).

The patient demographics will be reported with descriptive statistics.

The parameters between SMILE Xtra and control group were compared with Mann-Whitney *U* test for age, Fisher's exact test for gender, and linear mixed model analysis for other parameters to account for the effect of both eyes from the same patients. *p* < 0.05 was considered statistically significant.

## 3. Results

A total of 21 SMILE Xtra eyes (12 patients) and 32 SMILE eyes (18 patients) were included for analysis. All eyes aimed for a plano correction. The average age was 28.1 ± 6.1 in SMILE Xtra and 27.5 ± 5.2 in control (*p* = 0.966). The baseline parameters of both groups were shown in Table 1. Both groups had comparable age, gender distribution, spherical equivalent refraction (SEQ), and mean keratometry. The SMILE Xtra group had significantly lower central corneal thickness. The RSB thickness was 288 ± 33 *μ*m in SMILE Xtra and 311 ± 30 *μ*m in control (*p* < 0.008). 14 SMILE Xtra eyes (66.7%) had an ectasia risk factor score of 4 or higher (high risk) and 4 eyes (19.0%) had a score of 3 (moderate risk). In the control group, 5 eyes (15.6%) were of high risk and 5 eyes (15.6%) had moderate risk.

### 3.1. Visual Acuity, Safety, and Efficacy

**Figure 1:**
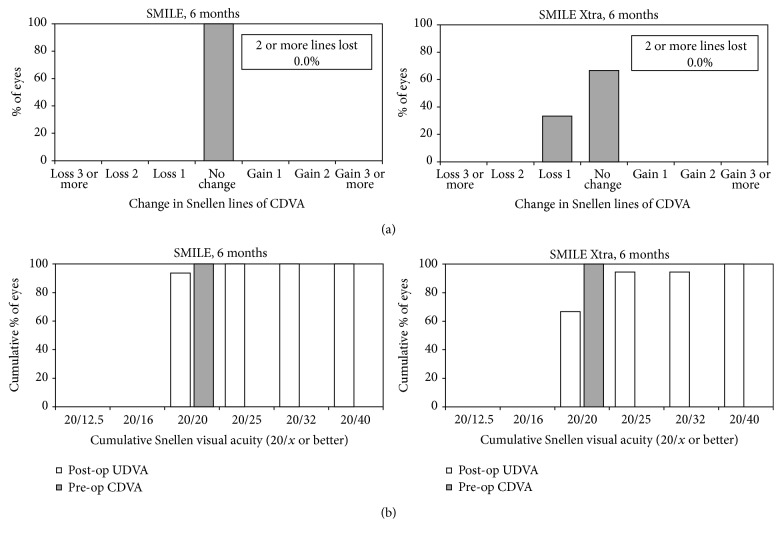
Visual outcomes for small-incision lenticule extraction (SMILE) (left) and combined SMILE with cross-linking (SMILE Xtra) (right) at the end of 6 months, showing the (a) change in preoperative and postoperative corrected distance visual acuity (CDVA) and (b) the cumulative percentage of eyes attaining specified cumulative levels of postoperative uncorrected distance visual acuity (UDVA).

For CDVA, no eyes in either group had lost 2 or more lines at all visits till postoperative month 6 (Figure 1). Six eyes (33%) lost 1 line of CDVA in the SMILE Xtra group while no eye had lost CDVA in the control group. At 6 months, only 67% eyes in SMILE Xtra group achieved UDVA 20/25 or better, while all except 2 eyes (94%) in the control group had UDVA 20/25 or better (Figure 1).

The safety index and efficacy index of both groups at months 1, 3, and 6 were shown in Table 2. Both indices were statistically significantly lower in the SMILE Xtra group than control at all visits. No intraoperative or postoperative complications occurred in either group. Postoperatively, there was no clinically detectable corneal haze in all cases.

### 3.2. Refraction, Predictability, and Stability

**Figure 2:**
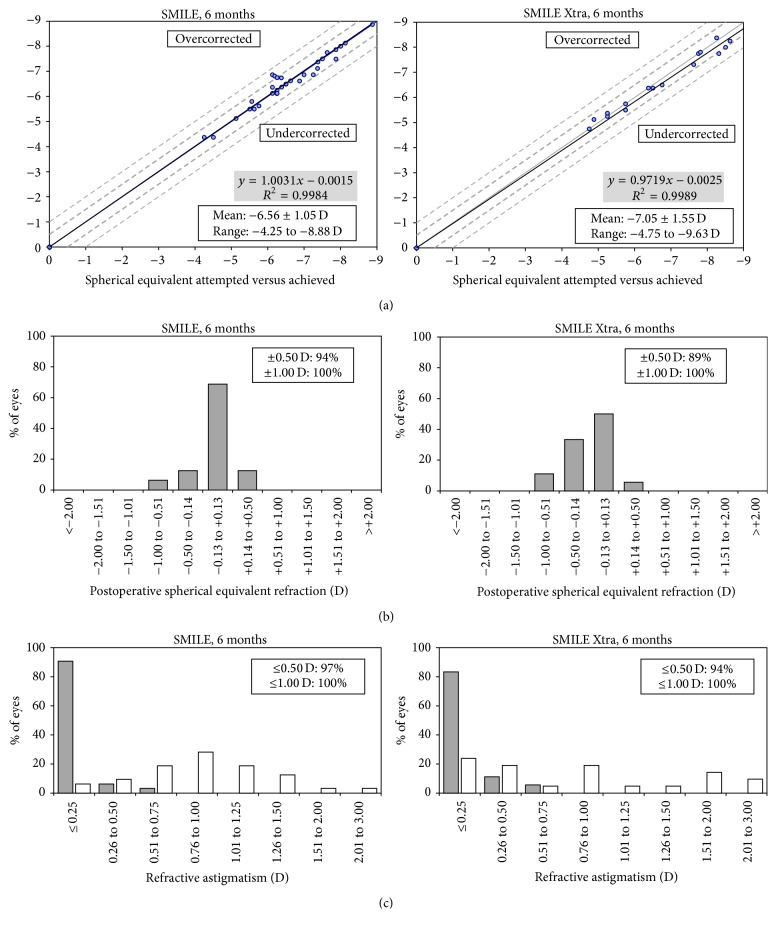
Refractive outcomes for small-incision lenticule extraction (SMILE) and combined SMILE with cross-linking (SMILE Xtra) at the end of 6 months postoperatively: (a) attempted versus achieved manifest spherical equivalent refraction (SEQ) correction in diopters for SMILE (left) and SMILE Xtra (right); (b) percentages of eyes within different diopter ranges of the intended correction in SEQ after SMILE and SMILE Xtra; (c) percentage of eyes attaining specified levels of astigmatism before (white) and after (grey) SMILE and SMILE Xtra.

**Figure 3:**
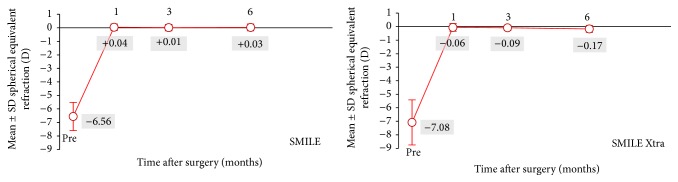
Time course of manifest spherical equivalent refraction (SEQ) after small-incision lenticule extraction (SMILE) and combined SMILE with cross-linking (SMILE Xtra).

The mean spherical equivalence reduced from −7.08 D to −0.17 D at 6 months postoperatively in SMILE Xtra and from −6.56 D to +0.03 D in control. At 6 months, 94% and 97% of eyes in the SMILE group were maintained within ±0.50 D in the SEQ and astigmatism correction, respectively. In SMILE Xtra group, 89% and 94% of eyes were within ±0.50 D in SEQ and astigmatism correction, respectively (Figure 2). The mean errors in the correction in the SEQ at 6 months were −0.17 ± 0.26 D for SMILE Xtra and +0.03 ± 0.25 D for control (*p* = 0.021).  Figure 3 shows that a slight myopic shifting trend is demonstrated in the SMILE Xtra group while the SMILE group demonstrated stability over 6 months.

### 3.3. Corneal Thickness

The baseline CCT and RSB thickness were thinner in the SMILE Xtra group. However, at 6 months postoperatively, the CCT in SMILE Xtra group was 420 ± 39 *μ*m, which was similar to control (408 ± 32 *μ*m, *p* = 0.507).

## 4. Discussion

Studies have demonstrated that SMILE is a safe procedure and has comparable outcome with femtosecond-LASIK [1, 3, 4, 6–8]. There is still debate whether SMILE is biomechanically stronger when compared with LASIK. Sinha Roy et al. proposed that SMILE might present less biomechanical risk to the residual bed of susceptible corneas than LASIK based on finite-element analysis [18]. Two clinical studies found less reduction in biomechanical properties measured with ORA after SMILE compared with LASIK [19, 20], especially in eyes with myopia more than 6 diopters. On the other hand, Pedersen et al. and Sefat et al. reported similar reduction in biomechanical properties measured with Corvis ST and ORA after LASIK or SMILE [21, 22]. From current evidence, it was still inconclusive whether the cornea after SMILE is biomechanically stronger than after LASIK. Theoretically, the cornea after SMILE is stronger than LASIK due to less disruption of the collagen fibers in the anterior stroma. However, the biomechanical strength is inevitably reduced after any corneal laser refractive procedures. The use of simultaneous cross-linking to restrengthen or compensate for the corneal biomechanical strength has therefore been proposed, especially together with LASIK (LASIK Xtra).

In view of the weakening effect on the stroma after LASIK, the use of LASIK Xtra had gained popularity in the past few years and has reported good refractive and topographic stability comparable with LASIK alone [12–15]. However, there was still a lack of standardized procedure protocol and patient selection criteria. Various irradiation protocols have been reported, and the UVA energy dose ranged from 1.35–5.4 J/cm^2^. The rationale for adding simultaneous crosslinking for the patients in our SMILE Xtra group was similar to LASIK Xtra. Our SMILE Xtra eyes had a significantly thinner CCT and RSB thickness than the control, with an increased risk of ectasia. We applied the riboflavin directly to the stromal pocket after lenticule extraction, followed by UVA irradiation with a total energy of 0.8 J/cm^2^ (18 mW/cm^2^ for 45 seconds). This method of performing cross-linking was in fact very similar to the concept of intrastromal cross-linking in treatment of keratoconus, where a corneal stromal tunnel was created using femtosecond laser (either alone or in conjunction with intrastromal corneal ring segment implantation) [23, 24]. Intrastromal crosslinking with a pocket depth of 70 to 100 *μ*m and total energy ranging from 3.6 to 6.3 J/cm^2^ have been shown to be safe and effective in stabilizing keratoconus [23, 24]. However, results from animal studies on the biomechanical effect were inconclusive [25, 26]. There was no standardized protocol in the irradiation intensities and durations in the literature. Similarly, the optimal dose of delivering the UVA in SMILE Xtra is yet to be determined [27, 28]. Graue-Hernandez et al. performed SMILE Xtra on eyes with forme fruste keratoconus where they employed the standard Dresden protocol with 5.4 J/cm^2^ total energy, and good refractive outcomes were reported at two years [27]. Another recent study by Ganesh and Brar reported good safety outcome at 1 year after performing SMILE Xtra on at-risk (but not forme fruste keratoconus) eyes [28], where a protocol of 45 mW/cm^2^ for 75 seconds (total energy: 3.4 J/cm^2^) was used.

The UVA energy dose in our SMILE Xtra series (0.8 J/cm^2^) was lower compared with those used in other LASIK Xtra or SMILE Xtra protocols (1.35–5.4 J/cm^2^). Previously, we have employed a stronger protocol with 60 seconds' soak time and 60 seconds' irradiation time. We found around 10% of cases still had clinical observable haze at 6 months (unpublished data). As a prophylactic treatment, a good balance between corneal restrengthening and visual outcome is preferred. Therefore, in this study, we employed a lower-energy protocol to minimize the postoperative haze. Unfortunately, due to limitation of resources, our study did not perform biomechanical studies to study the effect of the cross-linking. For the clinical effect, one would expect long-term studies (in terms of years) are needed to prove the benefit of SMILE, which has been shown to be a relatively stable procedure. A recently published 5-year SMILE outcome study showed stable refraction in SMILE with 0.48 D regression over 5 years [29]. The reported regression over 5 years in LASIK ranged from 0.6 to 0.96 D [1, 30]. Therefore, the main purpose of this current study is to report the short-term visual and refractive outcome of SMILE Xtra with a low energy protocol. The other reason that we used a low energy protocol was to avoid a continued corneal flattening effect, which was reported in the treatment of keratoconus using the usual energy dose (5.4 J/cm^2^) [31]. This continued corneal flattening effect is desirable in keratoconus treatment, but not in refractive surgery in healthy eyes. Since the energy dose we employed was much lower, the flattening effect was expected to be much less. We put emphasis on striking a balance between prophylaxis and good visual outcome.

Our SMILE Xtra results reported a high level of refractive predictability. At 6 months, 89% of our SMILE Xtra eyes were within ±0.50 D from target and 100% were within ±1.00 D, and this was also comparable with refractive predictability reported in the literature [4, 5]. However, both our SMILE Xtra group and their results had a small undercorrection at 6 months. This could be explained by a lack of normogram adjustment for SMILE Xtra. This slight undercorrection also explained the small difference in the efficacy index between our SMILE Xtra and control, where at 6 months only 67% SMILE Xtra eyes had UDVA reaching 20/20, compared with 94% in control.

In our SMILE cases, the safety index at 3 and 6 months postoperatively was 1.00. This was comparable to the largest reported series by Ivarsen et al., where their safety index at 3 months was 1.05 [2]. In our SMILE Xtra cases, the safety index was slightly lower at 0.95 and 0.96 at 3 and 6 months, respectively. Nevertheless, no eyes in our series lost more than 1 line of CDVA, and this was the same as the SMILE Xtra series reported by Ganesh and Brar [28], where no eyes lost more than 1 line of CDVA either. This difference could be explained by subclinical corneal stromal haze induced by cross-linking in our SMILE Xtra eyes. Moreover, since the stromal interface was irrigated with riboflavin solution and cross-linked, the wound healing response at the stromal interface could be altered compared with SMILE alone.

A limitation of the current work was unable to perform direct biomechanical measurements due to limited resources. Nevertheless, it had been shown that direct in vivo measurement of the corneal biomechanical property had low specificity and sensitivity [32]. The slight myopic shift demonstrated in our SMILE Xtra group could as well be due to an inadequate CXL effect to strengthen the cornea of our SMILE Xtra cases (where the preoperative corneas are thinner). The strengthening effects of SMILE Xtra have been supported by an ex vivo study on human cornea by Kanellopoulos et al., which showed that combining cross-linking with refractive lenticule (5 mm diameter and 80 *μ*m thickness at 140 *μ*m depth) extraction resulted in significantly increased biomechanical properties compared with control non-cross-linked eyes [32]. We did not routinely check the endothelial cell count either, as it has already been shown that there was no significant change in the endothelial cell count before and after SMILE [33] or in SMILE Xtra [28]. Other limitations of this study included small sample size and a relatively short follow-up. Our center only had routine follow-up until 6 months postoperatively since it is a private practice setting. Therefore, we aim to report the early safety and predictability of this procedure only. Given the regression after SMILE was only 0.48 D over 5 years [29], we believe only long-term data is required to study the beneficial effect of SMILE Xtra compared with SMILE alone. This emerging technique has the potential to restore the biomechanical strength of the cornea after refractive procedures. We have proposed a low energy protocol to strike a balance between prophylaxis and visual outcome. However, more long-term research data are required to guide the optimal protocol of simultaneous cross-linking.

## Figures and Tables

**Table 1:** Baseline parameters of combined small-incision lenticular extraction (SMILE) and collagen cross-linking (SMILE Xtra) versus SMILE.

	SMILE Xtra	SMILE (control)	*p* value
Age	28.1 ± 6.1	27.5 ± 5.2	0.966^a^
Sex (male : female)	4 : 8	9 : 9	0.465^b^
Sphere (diopter)	−6.59 ± 1.55	−6.05 ± 1.09	0.124^c^
Cylinder (diopter)	−0.96 ± 0.72	−1.02 ± 0.47	0.567^c^
Spherical equivalent refraction (diopter)	−7.08 ± 1.67	−6.56 ± 1.05	0.167^c^
Mean keratometry (diopter)	43.71 ± 1.56	43.37 ± 1.28	0.396^c^
Central corneal thickness (*μ*m)	518 ± 39	545 ± 22	0.007^c*∗*^

^a^Mann-Whitney *U* test.

^b^Fisher's exact test, by subject.

^c^Linear mixed model, by eye.

^*∗*^ *p* < 0.05.

**Table 2:** The safety indices (postoperative CDVA/preoperative CDVA) and efficacy indices (postoperative UDVA/preoperative CDVA) of both groups at postoperative months 1, 3, and 6.

	Month 1	Month 3	Month 6
Safety index			
SMILE Xtra	0.95 ± 0.08	0.95 ± 0.06	0.96 ± 0.06
SMILE	1.03 ± 0.09	1.00 ± 0.04	1.00 ± 0.00
*p* value^*∗*^	0.004	0.005	<0.001
Efficacy index			
SMILE Xtra	0.89 ± 0.11	0.89 ± 0.10	0.88 ± 0.13
SMILE	0.96 ± 0.08	0.98 ± 0.07	0.97 ± 0.06
*p* value^*∗*^	0.013	0.001	0.005

^*∗*^Linear mixed model analysis.
